# Survival of a microbial inoculant in soil after recurrent inoculations

**DOI:** 10.1038/s41598-024-54069-x

**Published:** 2024-02-20

**Authors:** M. Papin, L. Philippot, M. C. Breuil, D. Bru, A. Dreux-Zigha, A. Mounier, X. Le Roux, N. Rouard, A. Spor

**Affiliations:** 1 Univ Bourgogne Franche Comte, INRAE, Institut Agro Dijon, Agroecologie, 17 Rue Sully, 21000 Dijon, France; 2 GreenCell Biopole Clermont Limagne, 63360 St Beauzire, France; 3 Universite Claude Bernard Lyon 1, Microbial Ecology Centre LEM, INRAE, CNRS, VetAgroSup, UMR INRAE 1418, 43 Blvd 11 Novembre 1918, 69622 Villeurbanne, France

**Keywords:** PGPR, Nitrogen cycle, Soil bacteria, Recurrent inoculation, Microbial ecology, Soil microbiology

## Abstract

Microbial inoculants are attracting growing interest in agriculture, but their efficacy remains unreliable in relation to their poor survival, partly due to the competition with the soil resident community. We hypothesised that recurrent inoculation could gradually alleviate this competition and improve the survival of the inoculant while increasing its impact on the resident bacterial community. We tested the effectiveness of such strategy with four inoculation sequences of *Pseudomonas fluorescens* strain B177 in soil microcosms with increasing number and frequency of inoculation, compared to a non-inoculated control. Each sequence was carried out at two inoculation densities (10^6^ and 10^8^ cfu.g soil^−1^). The four-inoculation sequence induced a higher abundance of *P. fluorescens*, 2 weeks after the last inoculation. No impact of inoculation sequences was observed on the resident community diversity and composition. Differential abundance analysis identified only 28 out of 576 dominants OTUs affected by the high-density inoculum, whatever the inoculation sequence. Recurrent inoculations induced a strong accumulation of nitrate, not explained by the abundance of nitrifying or nitrate-reducing microorganisms. In summary, inoculant density rather than inoculation pattern matters for inoculation effect on the resident bacterial communities, while recurrent inoculation allowed to slightly enhance the survival of the inoculant and strongly increased soil nitrate content.

## Introduction

Synthetic fertilisers and pesticides have played an essential role in achieving contemporary agricultural yields. However, the continuous and excessive application of synthetic fertilizers and pesticides is a growing concern due to their potential adverse effects on human health and ecosystems^1–3^. Microbial inoculants represent promising sustainable alternatives to agrochemical inputs as they offer benefits in terms of crop yield and nutrient use efficiency^4^, plant tolerance to biotic and abiotic stresses^5^, resistance to pathogens^6^ and adaptation to climate change^7^. This growing interest in microbial inoculants over the last decade is reflected both by an increase in studies on the subject^8^ and by substantial investments for their development^9^. Nevertheless, translation of their beneficial effects from controlled conditions to field applications presents many challenges^10^ including their unreliable survival in soil following application^11^. Thus, numerous studies have reported a steady decline of inoculants in soil after application with their persistence depending on a variety of biotic and abiotic factors and thus severely impacting their efficacy^12,13^.

Invasion ecology focuses on the factors that allow invasive species to survive and thrive in a new environment as well on their impact on native ecosystems. As such, an increasing number of studies are adopting an invasion ecology approach to unravel the mechanisms underlying the survival of microbial inoculants. In this context, a microbial inoculant is considered as an alien species introduced into a resident community which has to overcome both abiotic and biotic hurdles before its establishment^14^. Soil type and diversity are known to affect the invasibility of an environment^15^. Specifically, a greater diversity of the resident community along with the presence of closely related species can increase the propensity of niche overlap between the soil community and the invader, therefore hampering invasion as suggested by the Darwin’s naturalisation hypothesis^16–20^. On the contrary, it has also been posited that invaders closely-related to resident species would be more likely to establish due to shared pre-adaptation with resident species^21,22^.

While diversity and composition of the resident community modulate invasion success, invaders generally affect this resident community in return^23,24^, with both compositional and functional changes, including shifts in nitrogen cycling^25–27^. In addition, the impact of microbial inoculations on the resident soil microbial communities has often been overlooked despite calls for a more comprehensive assessment of the consequence of microbial inoculation^28,29^. Mallon et al.^30^ showed that even transient and unsuccessful bacterial invasion can cause changes in the diversity, structure and niche breadth of the invaded bacterial community, and they specifically observed a shift in the community niche away from the resources used by the invader. According to Darwin’s naturalisation hypothesis, this shift could in turn favour further invasions by the same strain as the competition for resources will be less intense as hypothesised by Mallon et al.^30^.

In this work, we sought to assess to which extent inoculation frequency is affecting the inoculant survival, as well as its impact on the resident soil microbial community. We tested the hypothesis stated by Mallon et al.^30^ that recurrent inoculations of the same strain would affect the resident community and enhance the survival and establishment of the inoculant. To test this hypothesis, we inoculated soil microcosms with *Pseudomonas fluorescens* B177 at two densities with four sequences of inoculation ranging from one to four inputs compared with a non-inoculated control. We then monitored the survival of the inoculated strain and assessed the impact of recurrent inoculations on the composition and diversity of the resident bacterial community as well as on microbial functions. The nitrogen cycle being an essential agrosystemic service in agriculture and a common indicator of soil quality^31^, we chose it as the model function in this study.

## Results

### Inoculant survival following single or recurrent inoculations

**Figure 1:**
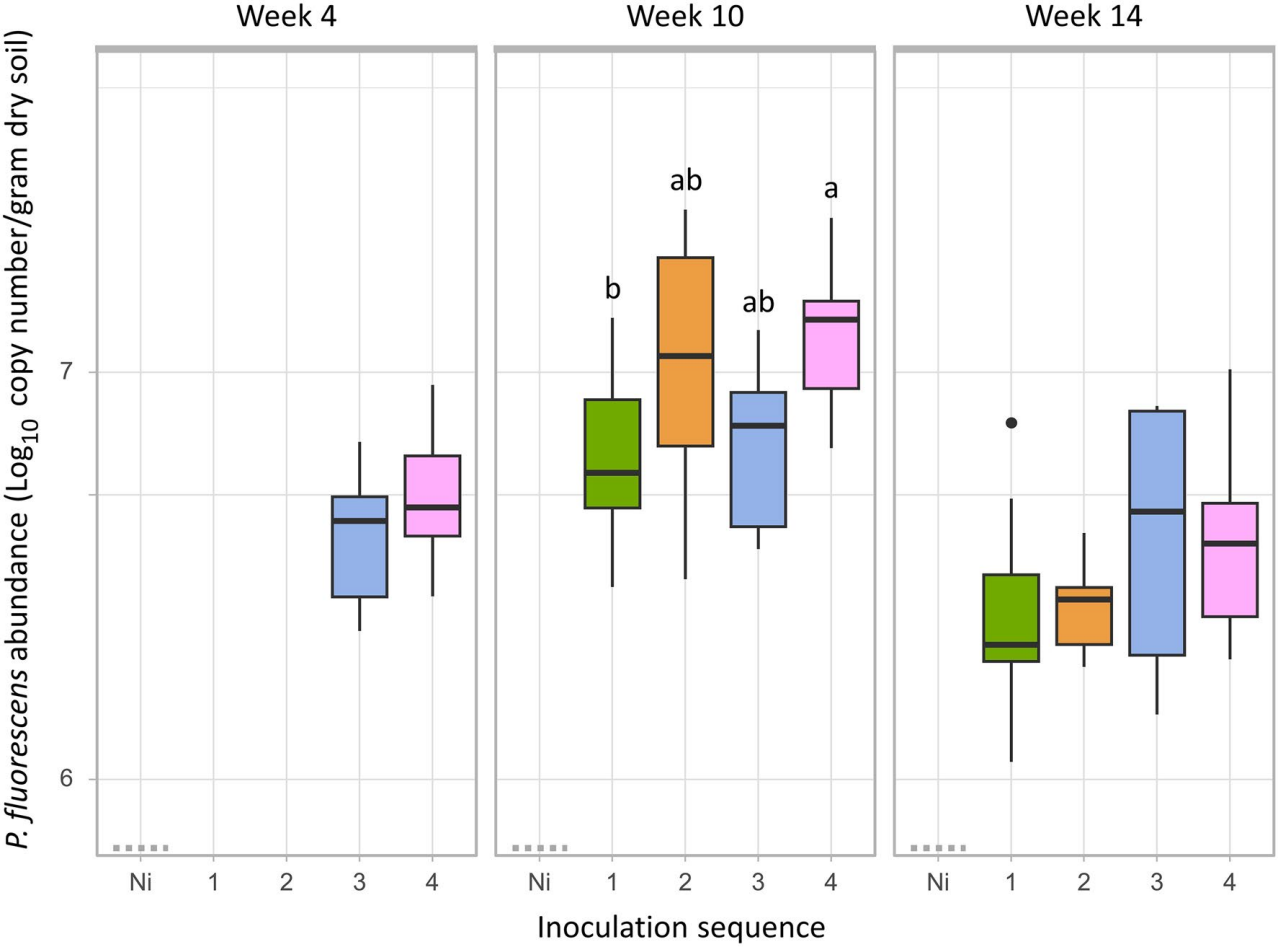
Survival of the inoculant. Abundance of Pseudomonas fluorescens across inoculation sequences (1, 2, 3 and 4) over time (week 4, 10 and 14) for high inoculum density (10^8^ cfu g dry soil^−1^). Values represent the log copy number per gram of dry soil. Letters above the bars indicate significant differences between sequences for each time block according to Tukey’s test (p = 0.03).

*Pseudomonas fluorescens* was not detected in the non-inoculated controls. In 55% of the low inoculum density samples (10^6^ cfu g^−1^ dry soil), the strain was detected but under the quantification limit while it was always above the quantification limit in the high inoculum density samples (10^8^ cfu g dry soil^−1^). Therefore, only the high inoculation treatment was included in the quantitative analysis. *P. fluorescens* abundance was up to 1.3 × 10^7^ and remained above 10^6^ copy g^−1^ dry soil in all inoculation sequences until the end of the experiment. At week 10, *P. fluorescens* abundance was significantly higher in Sequence 4 (four inoculations) compared to Sequence 1 (one inoculation) (Tukey’s test, p = 0.03, Fig. 1). However, this difference was not significant anymore at week 14, corresponding to a transient effect of the recurrent inoculations. The abundances of *P. fluorescens* in Sequences 2 and 3 (two inoculations, 2 or 6 weeks apart) were not significantly different from that in Sequences 1 and 4.

### Diversity and composition of the indigenous bacterial community

**Figure 2:**
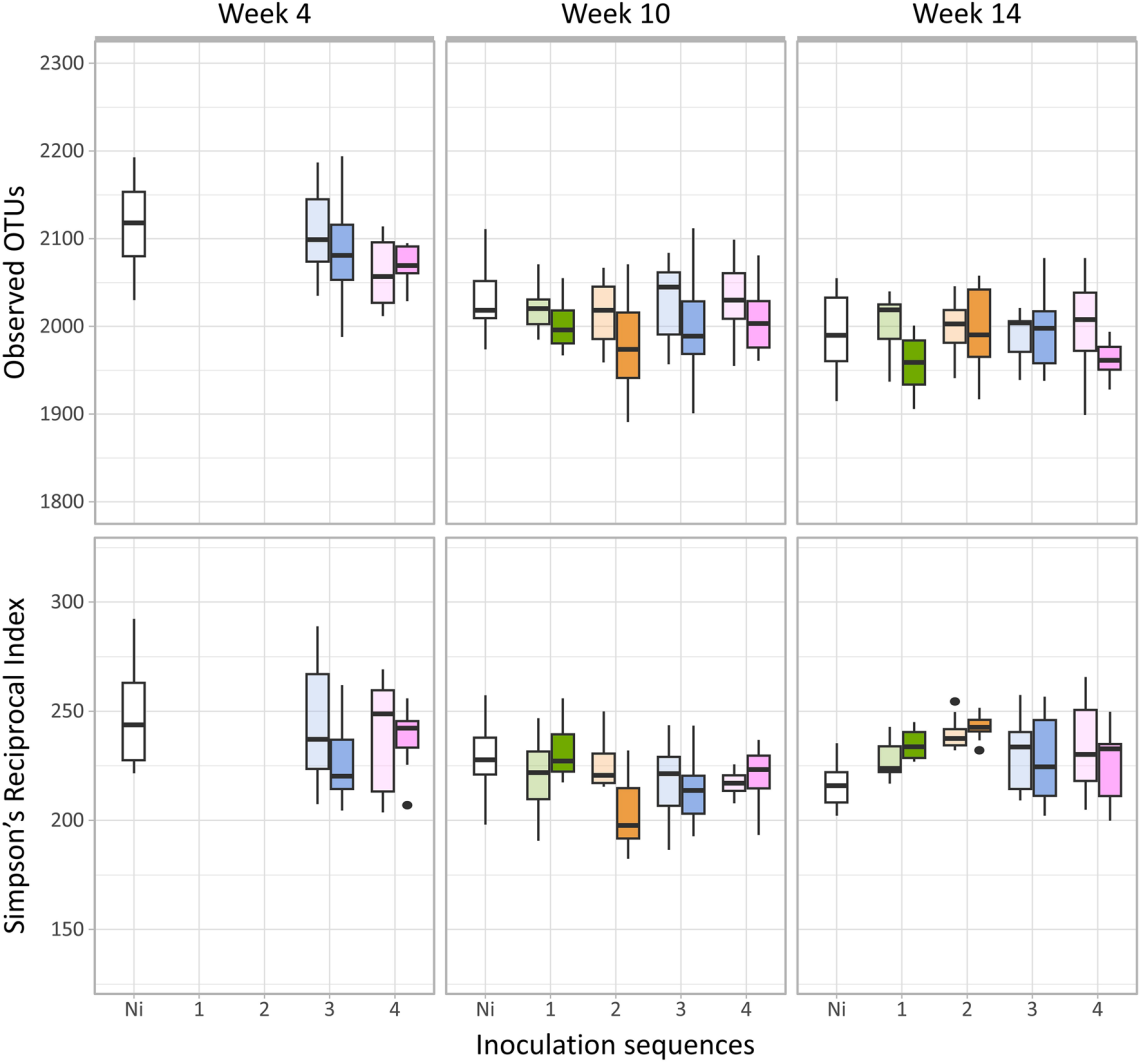
Diversity across the inoculation sequences. Number of observed OTUs and Simpson’s Reciprocal index at week 4, 10 and 14 for the four inoculation sequences and NI control. Bright colour stands for high density of inoculation (10^8^ cfu g dry soil^−1^) and light colour for low density of inoculation (10^6^ cfu g dry soil^−1^). Differences between groups were tested using ANOVAs followed by Tukey’s honestly significant difference test (p value < 0.05).

Analysis of the alpha diversity of the resident soil bacterial community showed no significant differences between the non-inoculated and inoculated soils (Fig. 2). However, high density inoculations resulted in a significant decrease of the richness, estimated as the number of observed OTUs (Tukey; p < 0.001), at both weeks 10 and 14, whereas no effect was observed on the Faith’s PD and Simpson reciprocal index (Supplementary Fig. 2a,b). While the Simpson reciprocal index remained stable in the non-inoculated treatment, a higher Simpson reciprocal index was observed at week 14 compared to week 10 in the inoculated microcosms (Tukey; p = 0.01, Supplementary Fig. 2c).

We found a significant though weak effect of the inoculation density on the structure of the bacterial community (PERMANOVA nperm = 999, R^2^ = 0.014, p = 0.017) and no effect of the inoculation sequence (PERMANOVA nperm = 999, R^2^ = 0.012, p = 0.22) based on both weighted and unweighted UniFrac distances (Supplementary Fig. 3). Thus, the structure of the bacterial community differed significantly between low and high inoculum density without any difference compared to the non-inoculated treatment (PERMANOVA nperm = 999, R^2^ = 0.043, p = 0.001).

### Impact of the inoculation sequence treatments on the relative abundance of autochthonous OTUs

**Figure 3:**
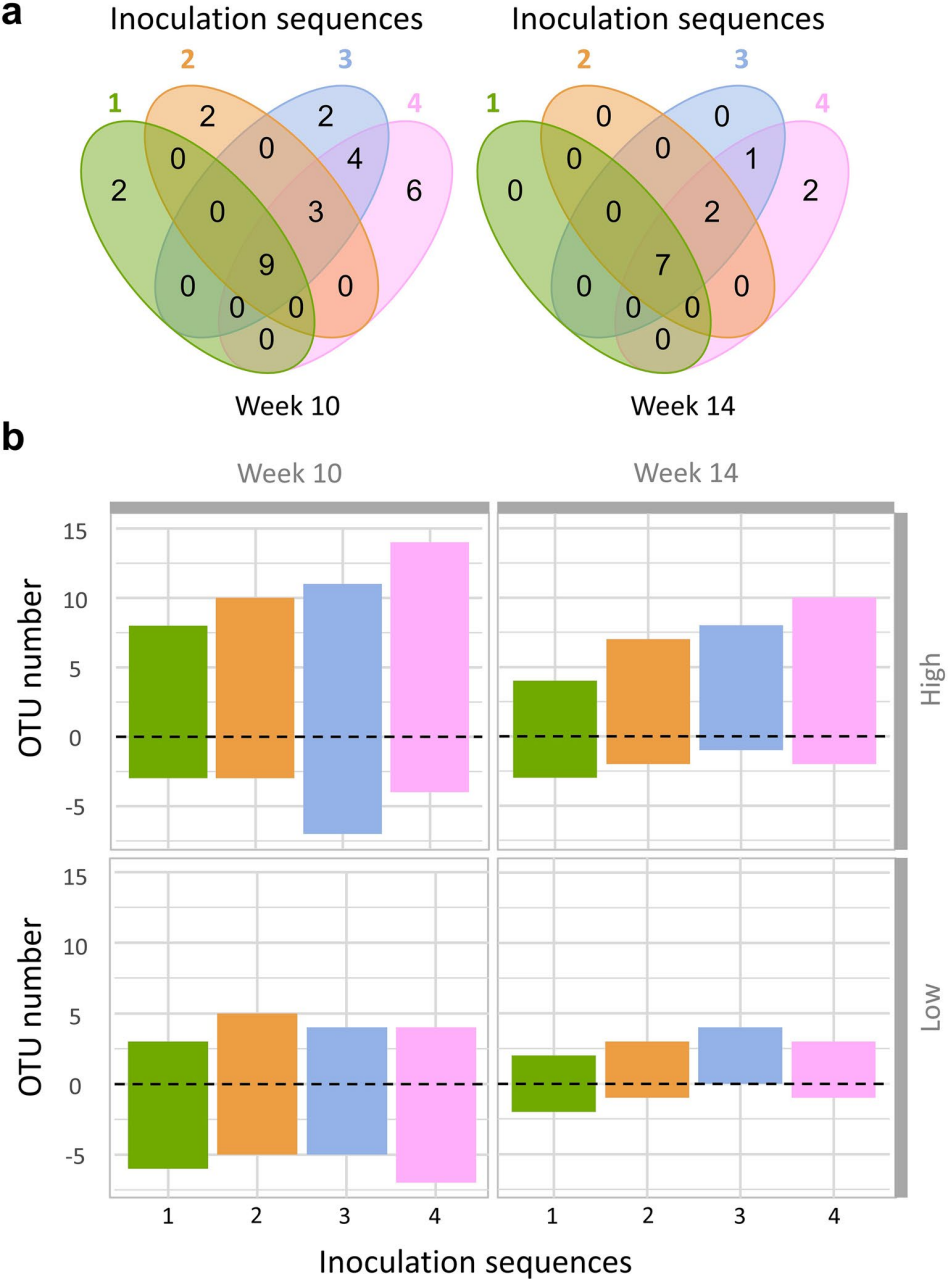
Number of OTUs affected by each inoculation Sequence. (**a**) Venn diagrams indicate the number of OTUs whose abundance is significantly different from the NI control for each inoculation sequence at week 10 and week 14 after high inoculation density (10^8^ cfu g dry soil^−1^). (**b**) The changes in abundances whether positive or negative compared to the NI control for all inoculation sequences and both high and low inoculation density (10^8^ and 10^6^ cfu g dry soil^−1^ respectively).

For a deeper insight into the effect of inoculations on the resident bacterial taxa, we used a generalised linear mixed model to identify OTUs exhibiting significant differences in relative abundance between the inoculated treatments and the non-inoculated control (p < 0.05). Only 28 out of the 576 dominant OTUs were significantly affected by *P. fluorescens* across the different inoculation sequences at week 10. While 9 of the impacted OTUs are shared by all inoculation sequence treatments, sequences 3 and 4 also affected 9 and 13 other OTUs as compared to 2 and 5 other OTUs only affected in the Sequences 1 and 2 (Fig. 3a). The total number of impacted OTUs decreased at week 14 (12 OTUs affected) with once again an increasing number of impacted OTUs from Sequence 1 to 4. Interestingly, a majority of OTUs significantly affected by inoculation exhibited increased relative abundances, especially in the high-density treatments (Fig. 3b).

For example, at week 10, 16 OTUs exhibited increases in relative abundance compared to the non-inoculated control whatever the inoculation sequence while, at the same time, 7 OTUs were negatively affected. Most of these impacted OTUs belonged to the Gammaproteobacteria phyla (38.5%) and one of them was assigned to *Pseudomonas fluorescens* (OTU 9). Interestingly, this OTU 9 exhibited a 99% sequence identity with the inoculated *Pseudomonas fluorescens* B177 strain. Its relative abundance increases with recurrent inoculations and was strongly and significantly correlated to the abundance of *P. fluorescens* B177 measured by qPCR (Pearson’s *r* = 0.88, p < 10^–16^) (Supplementary Fig. 4).

### Recurrent inoculation has a strong influence on the NO_3_^−^ pool

**Figure 4:**
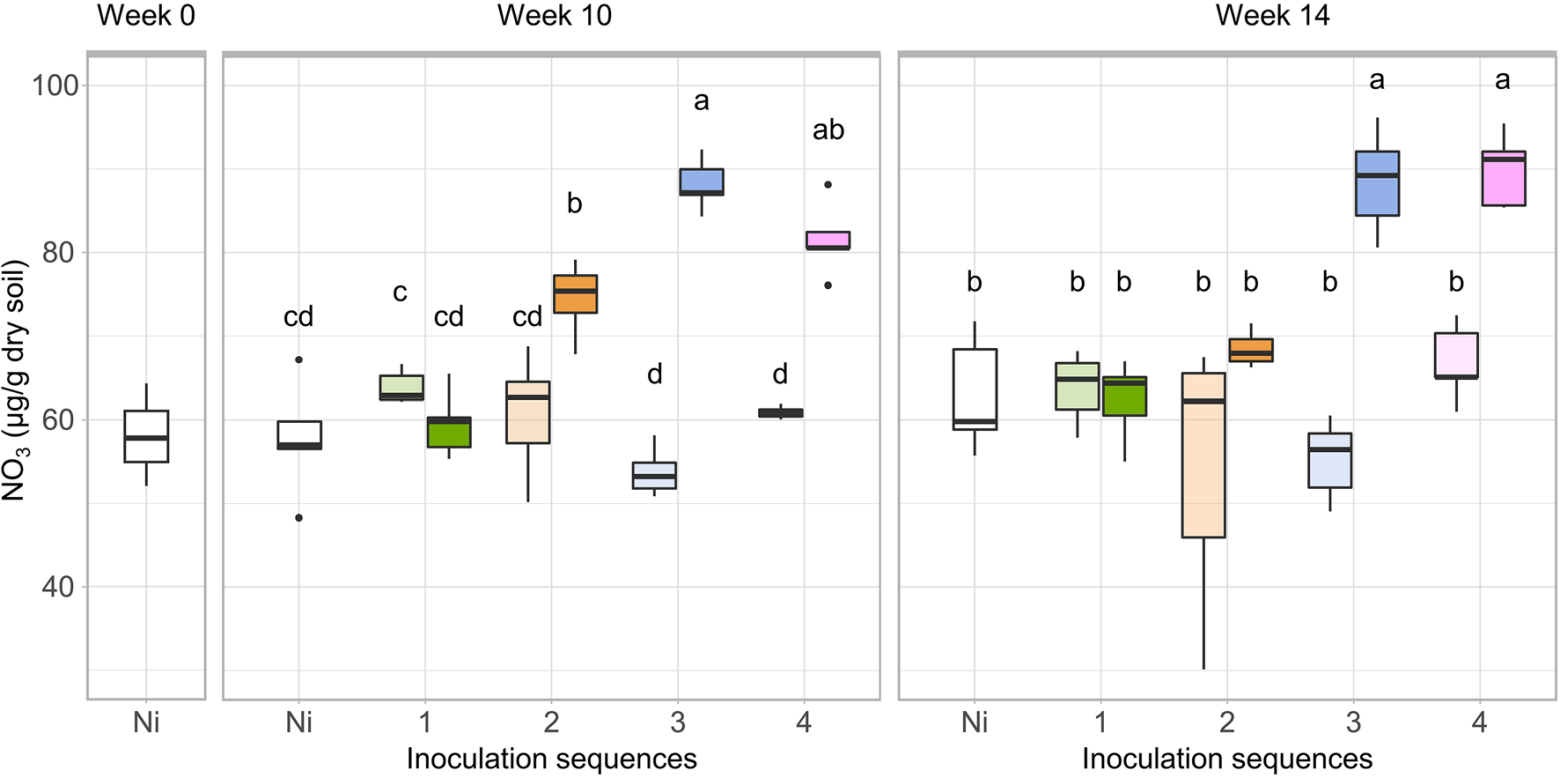
Nitrate accumulation in soil. Nitrate content (µg NO_3_^−^ g dry soil^−1^) at weeks 0, 10 and 14 for the four inoculation sequences and Ni control, after low (light colour) and high (bright colour) inoculation density. Letters above indicate significant differences between inoculation density and sequences according to Tukey’s test (p ≤ 10^–10^ at both week 10 and 14).

Ammonium concentrations remained low at every sampling time (below 3.17 µg NO_3_^−^ g^−1^ dry soil) whereas nitrate concentrations exceeded 30 µg NO_3_^−^ g^−1^ dry soil at all timepoints. At week 10, we found a significant effect of the inoculation sequence at high inoculation density with an increase of NO_3_^−^ in Sequences 2, 3 and 4 compared to inoculation sequence 1 and NI control (Fig. 4, p = 10^–14^). At week 14, significantly higher NO_3_^−^ levels were still observed in the inoculation sequences 3 and 4 (p = 10^–10^) but no longer in Sequence 2. When comparing with NO_3_^−^ concentrations at week 0, the recurrent inoculation of *P. fluorescens* at high density led to an accumulation of NO_3_^−^ in the soil that persisted at least for 6 weeks after the last inoculation. In contrast, at low inoculum density, NO_3_^−^ concentrations were similar between non-inoculated and inoculated treatments.

### Abundances of functional genes involved in nitrogen cycling

**Figure 5:**
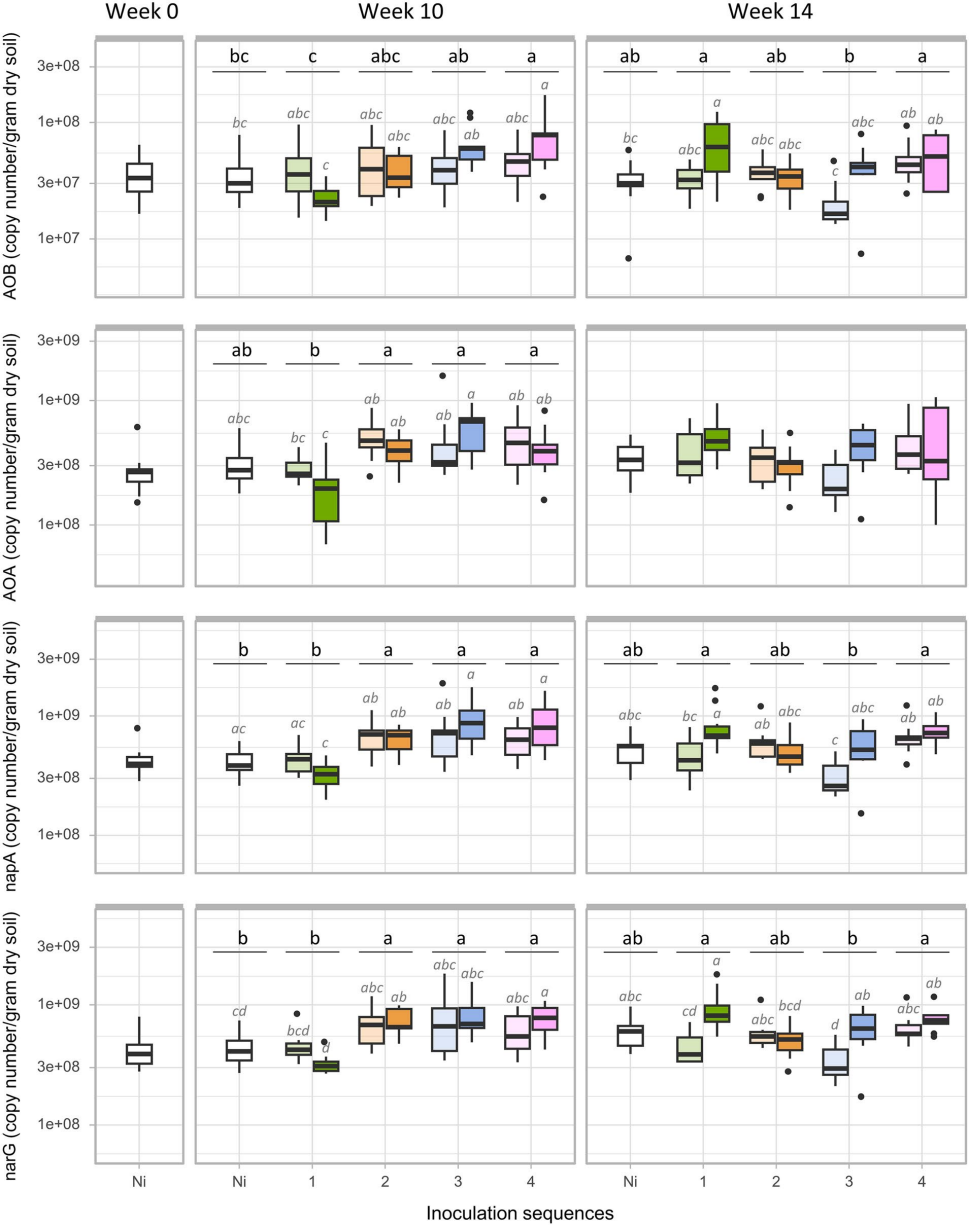
Functional impact of the inoculation sequences and density on the microbial community. Abundance of ammonia-oxidizers (AOB, AOA) and nitrate reducers (*napA* and *narG*) at weeks 0, 10 and 14 for the four inoculation sequences and Ni control, after low (light colour) and high (bright colour) inoculation density. Letters above indicate the significant differences between inoculation sequences (black letters) and a combined variable of inoculation sequence and density (grey letters) according to Tukey’s test (p < 0.005).

Quantitative real-time PCR assays were performed to quantify the abundances of 16S rRNA, bacterial *narG*, *napA* and *amoA* genes, as well as archaeal *amoA* genes as molecular markers for the microbial communities involved in NO_3_^−^ production and consumption. Inoculation density triggered only sporadic increases in the abundance of the *narG* and *napA* genes at week 10, however these increases were not significant when compared to the Ni control (Fig. 5). In contrast, the inoculation sequences induced more pronounced abundance changes with similar pattern across genes. At week 10, Sequences 2, 3 and 4 led to increased abundance of AOA, *napA* and *narG* as compared to Sequence 1, and also to the Ni control for *napA* and *narG*. Sequence 4 also increased AOB abundance at week 10 as compared to Sequence 1. At week 14, Sequences 1 and 4 increased the abundances of *narG*, *napA* and AOB as compared to Sequence 3 without significant difference compared to the control. We then investigated whether the increased NO_3_^−^ levels in the inoculation sequences 2, 3 and 4 were related to changes in the abundance of these N-cycling communities. However, we did not find any significant correlation.

## Discussion

Bacterial inoculation studies have often focused on the importance of the strain genotype^32^, root colonisation ability^33,34^, soil type^35^ or abiotic perturbations^36,37^ to identify the determinants of a successful inoculation. However, the resident microbial community also plays a crucial role in the fate of the inoculant^38^ and in turn, bacterial invasion can induce shifts in the resident microbial community that can influence the success of future inoculations^30^. Hence, previous inoculations could improve the maintenance capacity of a microbial inoculum in soil. Four strategies of recurrent inoculation were established at two inoculum densities to assess their effectiveness in promoting the survival of a bacterial inoculant and to determine the extent of their impact on the resident bacterial soil community.

### Inoculum density rather than recurrent inoculation improved the survival of the inoculant

At low inoculum density, the abundance of *P. fluorescens* B177 decreased with time to end up close to the detection limit with higher variability, which is inherent to the qPCR methodology at low copy number^39^. We therefore determined a quantification limit under which detection is possible but not quantification, as recommended in previous studies^40–42^. The high density of the bacterial inoculum used in our experiment exceeded field application by at least 5 orders of magnitude. For instance, commercial indication for *P. fluorescens* B177 inoculation is around 30 cells/g of soil under wettable powder form at 10^8^ viable cells/g, a common concentration to many other inoculant formulations^43,44^. Inoculation of *P. fluorescens* B177 at high density corresponded to 4.4% of the bacterial community (and 0.04% for the low density), which represents a substantial propagule pressure compared with the abundance of other OTUs. Propagule pressure, defined as the product of the number of invaders by the frequency of invasion events, has been shown to enhance the invasion success^45^ primarily due to a lower probability of stochastic extinction of the invaders^46^. Our study supports these findings since *P. fluorescens* abundance remained above 10^6^ copy/g of dry soil until week 14 when inoculated at high density while it declined below the detection limit at low inoculum density. Amor et al.^47^ demonstrated that a high propagule size favours invasion success when invaders exhibit a negative growth rate while a high propagule frequency is advantageous in the case of positive growth rate. This aligns with our study which shows a decline of *P. fluorescens* B177 over time, along with a much stronger effect of inoculum density than inoculum frequency. Yet, four recurrent inoculations (Sequence 4) transiently led to higher *P. fluorescens* abundance at week 10 at high inoculation density. This supports our main hypothesis that recurrent inoculation can improve the survival of the inoculum, but only the highest inoculation frequency was effective, and the benefit was rather limited.

### Diversity and composition of the resident bacterial community were weakly affected by recurrent inoculations

As bacterial invasions can affect the resident soil microbial community^23^, we assessed the impact of recurrent inoculation on the soil bacterial community. Previous studies have shown that even transient or unsuccessful invasion can induce a shift in the resident community structure and niche^30,48^. We observed a limited impact of the inoculant on the diversity and composition of the soil bacterial community despite the inoculum densities used (10^8^ and 10^6^ cfu g soil^−1^, representing 4.4 and 0.04% of the community, respectively). The higher inoculum density caused a small but significant decrease in richness as well as an increase of evenness along with changes in the relative abundance of 28 dominant OTUs (Fig. 3). There is no consensus on the effect of inoculation on the diversity of soil microbial community, with some studies reporting a diversity loss^49^ while others observed an increase in diversity^30,50^ or no effect at all^51^. The observed increase in evenness could be explained by the release of nutrients due to the death of the inoculant and favouring the growth of less abundant taxa^14^. This is supported by the results of the differential abundance analysis showing that most affected OTUs increased in abundance following inoculation (Supplementary Fig. 3). The lower richness in the inoculated treatments could be due to the high proportion of the inoculant representing up to 2.3% of the community at week 10, which can displace numerous rare taxa that will no longer be included in a rarefied data set.

Numerous studies have assessed the impact of single microbial inoculation on the resident soil community^24^ but less is known about the impact of recurrent inoculations. Despite the fact that the recurrent inoculation also increased the number of invaders, we did not observe any significant effect of the recurrent inoculation sequences on either the composition or the diversity of the resident soil bacterial community. This means that subsequent inoculations in Sequences 2, 3 and 4 did not further affect bacterial diversity beyond that the first inoculation at high density already had. Wang et al.^52^ also reported that the first inoculation with a consortium of four bacterial strains plays a greater role in influencing the community than any subsequent inoculations. In contrast, some studies reported no impact of inoculation of different strains of *P. fluorescens* on the soil microbiome at community level^53,54^. Nevertheless, when looking at the population level, the relative abundance of some OTUs was significantly affected (28 out of 576 dominant OTUs) with more OTUs affected as the number and frequency of inoculations increased. However, this inoculation effect on very few OTUs rather than on the global resident community underscores its remarkable resistance to invasion by *Pseudomonas fluorescens* B177.

There are two opposing theories concerning the interactions between an invader and closely related species in the resident community. Competition-relatedness hypothesis suggests that invasion is restricted due to overlapping of resource use and shared predators and pathogens^55^. On the contrary, it has also been postulated that a higher relatedness between invaders and resident population can promote invasion through shared mutualists, facilitation and likelihood to thrive in similar conditions^56^. Interestingly, in our study, most affected OTUs showed increased relative abundances, suggesting that positive interactions occurred between *P. fluorescens* and some members of the resident communities which favoured their relative fitness. Notably, among all impacted OTUs, the sharpest increase (i.e., 107-fold increase in relative abundance) was observed for one OTU affiliated to *Pseudomonas fluorescens* species and having 99% identity with the 16S gene of *P. fluorescens* B177. Along with the strong correlation between this OTU and the qPCR detection of the inoculant, this strongly suggests that this OTUs corresponds to the inoculated strain. However, due to the methodological limitation related to the sequencing of partial 16S rRNA it is not possible to rule out the possibility that OTU 9 also comprises a few other *Pseudomonas fluorescens*.

### Soil nitrate concentration was strongly impacted by the recurrent inoculations

We investigated whether recurrent inoculations had an impact on soil microbial functions, focusing on nitrogen cycling. Significant and persistent effects were observed with inoculation Sequences 3 and 4 at high density which led to increased soil nitrate concentrations at both weeks 10 and 14. Similar finding have been reported in previous studies, where shifts in soil microbial activities such as chitinase, urease or phosphatase were observed in response to microbial inoculation^57–59^. It is unlikely that *P. fluorescens* B177 had a direct effect on nitrate concentration because its genome does not contain any of the genes required for ammonia-oxidation (*amoA*) or dissimilatory nitrate-reduction (*napA* and *narG*). Since we observed significant differences in nitrate concentrations between Sequences 2 and 3 at high density, this effect was not due to the amount of nutrients released by dead inoculant cells. However, we cannot exclude an indirect effect related to the periodicity of the recurrent release of nutrients by dead inoculant cells. Alternatively, our results also suggest that under recurrent inoculation, *P. fluorescens B177* indirectly influenced nitrate concentrations in soil through changes in resident microbial taxa. This is supported by the fact that higher nitrate concentrations were observed in inoculation Sequences 3 and 4, which also had the strongest impact on the relative abundances of resident OTUs. However, we did not observe a significant correlation between any of the genes encoding for nitrate reduction (*napA, narG*), ammonia oxidation (AOA, AOB) and the nitrate concentrations (Fig. 5). While rare taxa are essential to support some key functions such as methane-production, with their absence leading to a drop in gas production^60^, in our study, rare taxa were excluded from the differential abundance analysis and were overlooked in the qPCR analysis. Therefore, it is possible that they have played a disproportionate role in the observed nitrate accumulation as this function undergoes fine-tuned regulation.

## Conclusion

In this work, we found that despite a high inoculum density of *Pseudomonas fluorescens* B177, the impact of recurrent inoculation had only a weak impact on the autochthonous microbial community. Inoculation density was the main factor driving the survival of the inoculant as well as the diversity and composition of the soil resident community. Recurrent inoculation led to a transient increase of the abundance of the inoculant compared to single inoculation with only a minor impact on members of the resident community. This shows that an invasion by *Pseudomonas fluorescens* B177, even at high density, did not induce any important shift in the community, explaining that recurrent inoculation with this strain could not facilitate future invasions by the same strain. Interestingly, recurrent inoculation sequences induced a strong and persistent accumulation of nitrate in soil that could not be attributed to changes in abundances of the targeted nitrogen cycling microbial guilds. Further investigations are needed to understand the mechanisms by which these recurrent inoculation sequences are impacting nitrogen-cycling activity of the resident microbial community.

## Methods

### Experimental design

Soil was collected from a maize field under conventional agriculture, located in South West of France (64450 Garlède-Mondebat, France). It was characterised as a silty soil (22.2% clay, 66.0% silt and 11.8% sand) with 2.82% organic matter and a pH in water of 5.9. Soil microcosms were prepared by filling 125 mL plasma flasks with 80 g equivalent of dry soil and sieved at 4 mm. The plant growth promoting rhizobacteria *Pseudomonas fluorescens* strain B177 (produced by GreenCell, France) was used as bacterial inoculant. *P. fluorescens* B177 was grown at 28 °C in TSB culture medium up to OD of 2. Bacterial cells were then centrifuged at 6000 rpm for 3 min and washed 3 times with a saline solution (0.9% NaCl). The cell pellets were then resuspended to obtain two inoculum densities (10^6^ and 10^8^ cell g of dry soil^−1^). Four inoculation sequences were applied for each inoculum density, with an increasing number of inoculation events. Sequence 1: one inoculation at day 56 (i.e. week 8), Sequence 2: two inoculations at days 42 and 56 (i.e. weeks 6 and 8), Sequence 3: two inoculations at days 14 and 56 (i.e. weeks 2 and 8), Sequence 4: four inoculations at days 0, 14, 42 and 56 (i.e. weeks 0, 2, 6 and 8). Non-inoculated soils were used as controls. Each treatment (inoculum density * inoculation sequence) was replicated 10 times for a total of 280 microcosms (Supplementary Fig. 1). After each inoculation, the moisture content in soil microcosms was adjusted to 70% of water-holding capacity (WHC). Microcosms were then watered every 2 weeks to maintain a range of 50–70% WHC. The soil microcosms were covered with gauze and incubated at room temperature (ranging from 19 to 21 °C) in the dark during the 14 weeks of experiment.

The soil from ten replicate microcosms per treatment was collected at days 0, 28, 70 and 98 (i.e. weeks 0, 4, 10 and 14) and thoroughly mixed before the following analyses. The sampling at days 70 and 98 corresponds to 2 and 6 weeks after the last inoculation regardless of the inoculation sequence, respectively. Soil sub-samples were stored at – 20 °C until DNA extraction. Soil humidity was also measured on fresh soil sub-samples at each sampling date.

### DNA extraction

For each microcosm, about 250 mg equivalent dry soil were weighted and DNA was extracted with the DNeasy 96 PowerSoil Pro kit (Qiagen) according to manufacturer’s instructions. DNA extracts were collected into 100 µL of TE buffer and quantified using the Quant-iT™ PicoGreen^®^ dsDNA Assay Kit (Invitrogen™, France) following the manufacturer's instructions.

### Quantification of the abundances of *Pseudomonas fluorescens* and functional genes

The abundance of *Pseudomonas fluorescens* B177 abundance was assessed by real-time quantitative PCR (qPCR) using the SCAR primers BP1F (5′-TGCCTGACGCTGTGATACTT-3′) and BP1R (5′-GCAGAAGAGATGAACCCACC-3′). The limit of quantification was assessed by serial dilutions of *P. fluorescens* genomic DNA according to Waiblinger et al.^40^. A relative standard error below 30% throughout the replicates was considered as the threshold to determine the limit of quantification which was determined as 80 copies per reaction, corresponding to an average of 4 × 10^5^ copy/g dry soil in our study. Linearised pGEM-T (pGEM-T Easy Vector, Promega) plasmid containing a clone targeted gene from *P. fluorescens* B177 strain was quantified by QuantiFluor dsDNA system (Promega). A serial dilution ranging from 10^2^ to 10^8^ copies per reaction was used as standards. qPCR reactions were carried out using a ViiA7 (Life Technologies, USA) in 15 µl reactions containing 7.5 µL of Takyon Low ROX SYBR 2X Master Mix (Eurogentec, France), 0.5 µM of each primer, 250 ng of T4 gene 32 (QBiogene, France) and 2 ng per well of DNA template. The PCR cycling conditions included initial denaturation of 95 °C for 3 min followed by 40 cycles of 95 °C for 15 s, 60 °C for 30 s and 72 °C for 30 s. Two runs were performed for each qPCR assay. Melting curves were run to verify the amplification specificity. The results were then processed using QuantStudio software (v1.7.1, Applied Biosystems).

The abundances of total bacterial and total fungal communities and targeted N-cycle microbial guilds (see below) were estimated by real-time quantitative PCR (qPCR) assays. Total bacterial and fungal communities were quantified using 16S rRNA primers as described by Muyzer et al.^61^. Abundances of N-cycle microbial guilds were estimated using the *amoA* gene to quantify bacterial (AOB) and archaeal (AOA) ammonia-oxidizers, and the *narG* and *napA* genes to quantify nitrate reducers^62^. DNA extracts diluted at 1ng/µL (described above) were used as template. Linearised pGEM-T plasmids containing the appropriate targeted gene were used as standards. PCR efficiency for the different assays ranged from 78 to 97%. No template controls resulted in null or negligible values. Inhibition in qPCR assays was tested by mixing control plasmid DNA (pGEM-T Easy Vector, Promega, France) with either soil DNA extracts or water. No inhibition was detected with the amount of DNA used.

### 16S rRNA amplicons sequencing

Bacterial diversity and composition were analysed via Illumina sequencing of the 16S rRNA amplicons generated from all DNA extracts using a two steps PCR procedure as previously described^63^. The first step consisted in PCR amplification of the V3-V4 hypervariable region of the bacterial 16S rRNA gene using the following primers: U341F (5′-CCTACGGGRSGCAGCAG-3′) and 805R (5′-GACTACCAGGGTATCTAAT-3′). First step PCRs were performed twice for each sample and pooled. In the second step, 6 µL of pooled 16S rRNA amplicons were used as template for a second PCR with Nextera-indexed sequencing primers to add multiplexing index-sequences. SequalPrep Normalisation kit (Invitrogen) was used to normalize amplicon concentrations prior to pooling. Sequencing was performed on Illumina MiSeq (2 × 250 pb paired-end reads) using the MiSeq reagent kit v2.

### Sequence processing and bacterial community composition

Sequences obtained from the 240 samples were processed with an in-house developed Python pipeline (https://forgemia.inra.fr/vasa/illuminametabarcoding). Shortly, 16S rRNA amplicon sequences were assembled using PEAR^64^ with default settings. Further quality checks were conducted using the scripts from the QIIME pipeline^65^ and short sequences were removed (< 350 bp). Reference-based and de novo chimera detection as well as OTU clustering were performed using VSEARCH^66^ and SILVA reference database^67^. The identity threshold was set at 94% based on replicate sequencing of a bacterial mock community. Representative sequences for each OTU were aligned using INFERNAL and phylogenetic tree was constructed using Infernal (version 1.1.3)^68^. Taxonomy was assigned using BLAST^69^ and the SILVA reference database 132^67^. In total, 8,075,341 sequences were obtained and assigned to 7443 OTUs. α-diversity metrics (observed OTUs, Simpson’s reciprocal index and Faith’s Phylogenetic Diversity) were calculated in QIIME based on rarefied OTU tables (> 10,000 sequences per sample). Bray–Curtis, weighted and unweighted UniFrac dissimilarity matrices^70^ were also computed to detect variations in the structure of microbial communities.

### Mineral nitrogen pool

Soil mineral nitrogen (NH_4_^+^ and NO_3_^−^) was extracted from 7 g of fresh soil sub-samples with 35 mL of 0.5M K_2_SO_4_ solution, then shaken (1 h, 80 rpm at room temperature), filtered and kept frozen until quantification. Colorimetric analysis was performed on Tecan infinite M200 (Life Sciences) according to Anderson and Ingram^71^.

### Data analysis

All statistical analyses were performed with R (version 4.0.1). For each sampling date, differences in the microbial α-diversity, gene abundance and mineral nitrogen pools were tested using ANOVA followed by Tukey HSD test (p < 0.05). Normality and homogeneity of the distribution of residuals were checked and log-transformation were applied when necessary. Permutational multivariate analysis of variance (PermANOVA) was carried out on the unweighted UniFrac distance matrices using adonis2 function implemented in the vegan package^72^. Pairwise comparisons between treatments were implemented using the pairwise.adonis function from the pairwiseAdonis package (version 0.4). Before comparing the differential abundance of OTUs, low abundance OTUs were filtered, keeping only OTUs with an average relative abundance above 1% across all samples. For each treatment, OTUs present in less than 60% of the samples were also excluded. These filtering steps reduce the zero counts in the OTU table which can increase the number of false positives in the differential abundance analysis. Pairwise comparisons were conducted on the resulting 576 OTUs using the following General Linear Mixed Model (GLMM)^73^. Considering that the abundance Y of each OTU, in any *k* replicate of any *i* treatment, follows a Poisson distribution of parameter ∆ as Y ~ P(∆), we used the following equation:

1 $${\text{log}}(\Delta_{{{\text{ik}}}} ) = {\text{o}}_{{{\text{ik}}}} { + }\mu + \alpha_{{\text{i}}} + {\text{Z}}_{{{\text{ik}}}} ,{\text{ Z}}_{{\text{ik 1}}} {{ \le {\text{k}} \le {1}0}} {\text{iida}}^{\text{{b}}}\sim \mathcal{N} \left( {0,{{\rho}}^{{2}} } \right)$$

Here, *o* represents the offset for each sample calculated as the logarithm of the sample read sum, α is the effect of each treatment and *Z* is the random sampling effect modelling the data overdispersion. Such model combines a generalised linear model and a mixed model. The generalised linear model allows to infer linear regression from data that does not follow a normal distribution because abundance data typically follow a Poisson distribution. The mixed model includes both fixed effects (treatment effects) and random effects (sampling effects). Each sampling time was analysed separately. The treatment effect was encoded as a factor combining the inoculation densities and the sequences, with nine levels. P values were adjusted using the *p.adjust* function with the false discovery rate (*fdr)* method to correct for the high number of OTUs tested. Pairwise comparisons were carried out between Ni control and treatments. Comparisons were considered significant when p < 0.05.

### Supplementary Information

Supplementary Information.

## Data Availability

16S rRNA raw sequences were deposited at the NCBI under the accession number PRJNA1033835. Data that support the findings of this study is also available from the corresponding author upon reasonable request.
