# Supplementary material for: Survival of a microbial inoculant in soil after recurrent inoculations
Source: Sci Rep. 2024 Feb 20;14:4177. doi: 10.1038/s41598-024-54069-x (PMC10879113; doi:10.1038/s41598-024-54069-x)
Supplement: Supplementary file 1 — Supplementary Information. [file 41598_2024_54069_MOESM1_ESM.pdf]

SUPPLEMENTARY FIGURES

Survival of a microbial inoculant in soil after recurrent inoculations

M. Papin[1,] L. Philippot[1*,] MC Breuil[1], D Bru[1], A. Dreux-Zigha[2], A. Mounier[1], X. Le Roux[3], N. Rouard[1], A. Spor[1]

[1] Univ Bourgogne Franche Comte, INRAE, Institut Agro Dijon, Agroecologie, 17 Rue Sully, F-21000 Dijon, France

[2] GreenCell Biopole Clermont Limagne, F-63360 St Beauzire, France

[3] Universite Claude Bernard Lyon 1, Microbial Ecology Centre LEM, INRAE, CNRS, VetAgroSup, UMR INRAE 1418, 43 Blvd 11 Novembre 1918, F-69622 Villeurbanne, France

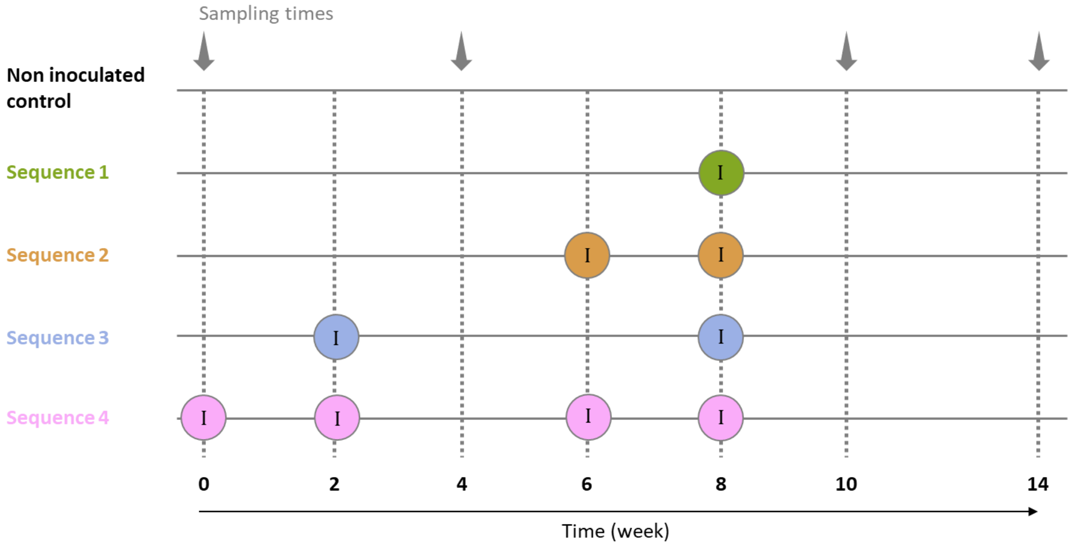

Supplementary Figure 1: **Schematic illustration of the experimental design.** Four sequences of inoculation were applied over time (green, orange, blue and pink circles). Each sequence was inoculated at two densities (10^6 and 10^8 cfu.g dry soil^{-1}). Each treatment (inoculation sequence x density) was replicated 10 times. A non-inoculated control was also performed in 10 replicates. Destructive sampling was performed on all replicates (grey arrows) before the following analysis: 16S rRNA sequencing, qPCR for P. f*luorescens* abundance, quantification of mineral nitrogen and qPCR for functional genes abundance (AOA, AOB, *napA* and *narG*).

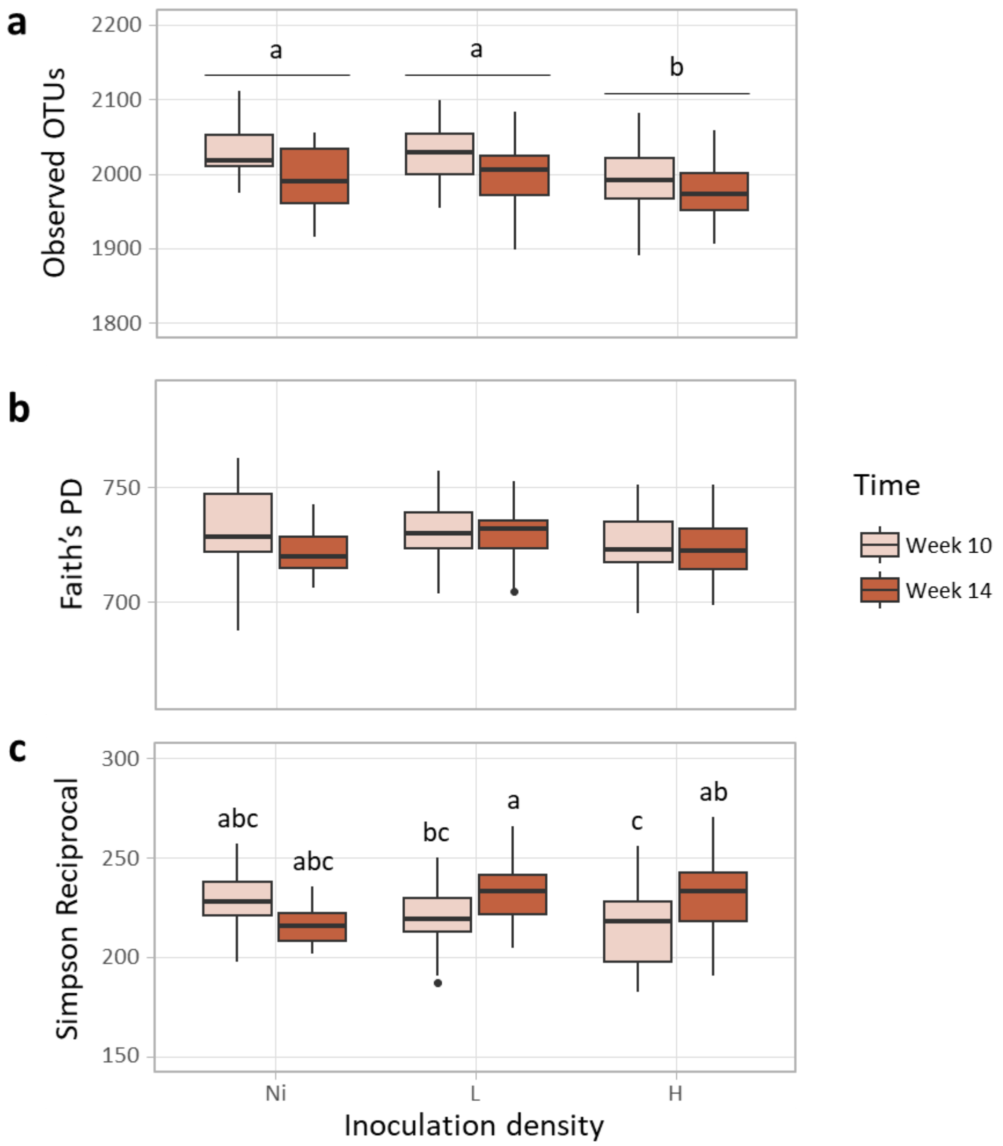

***Supplementary 2 : Diversity changes in the microbial community**. Observed OTUs (a), Faith's PD (b) and evenness expressed by Simpson reciprocal index (c) at week 10 and week 14 for the three inoculations density: non-inoculated (Ni), 10^6 cfu/g soil (L) and 10^8 cfu.g dry soil^{-1} (H). Letters above indicate significant differences according to Tukey's test (A: p-val = 10^{-5}, C: p-val = 0.01).*

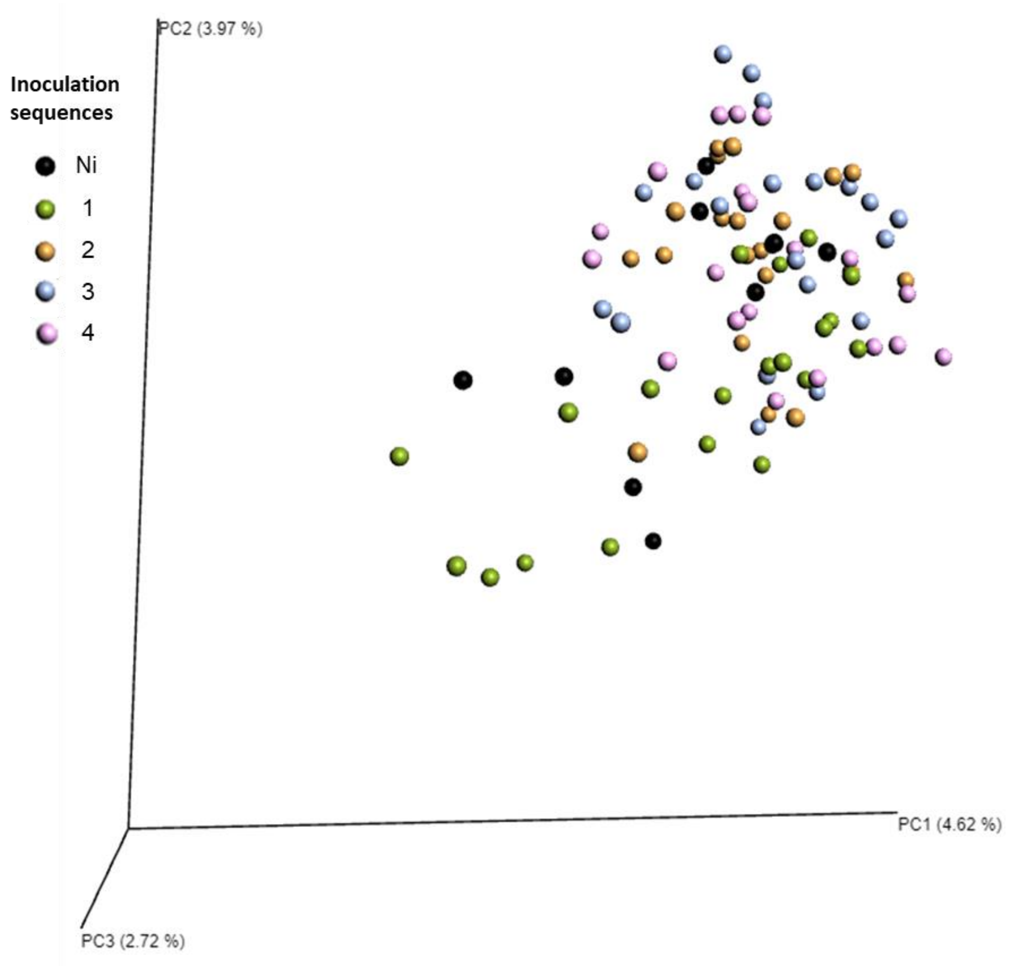

Supplementary 3 : Principal coordinate analysis based on Bray-Curtis dissimilarity. *Comparison of the distances between sequences of inoculation at week 10 for high inoculation density.*

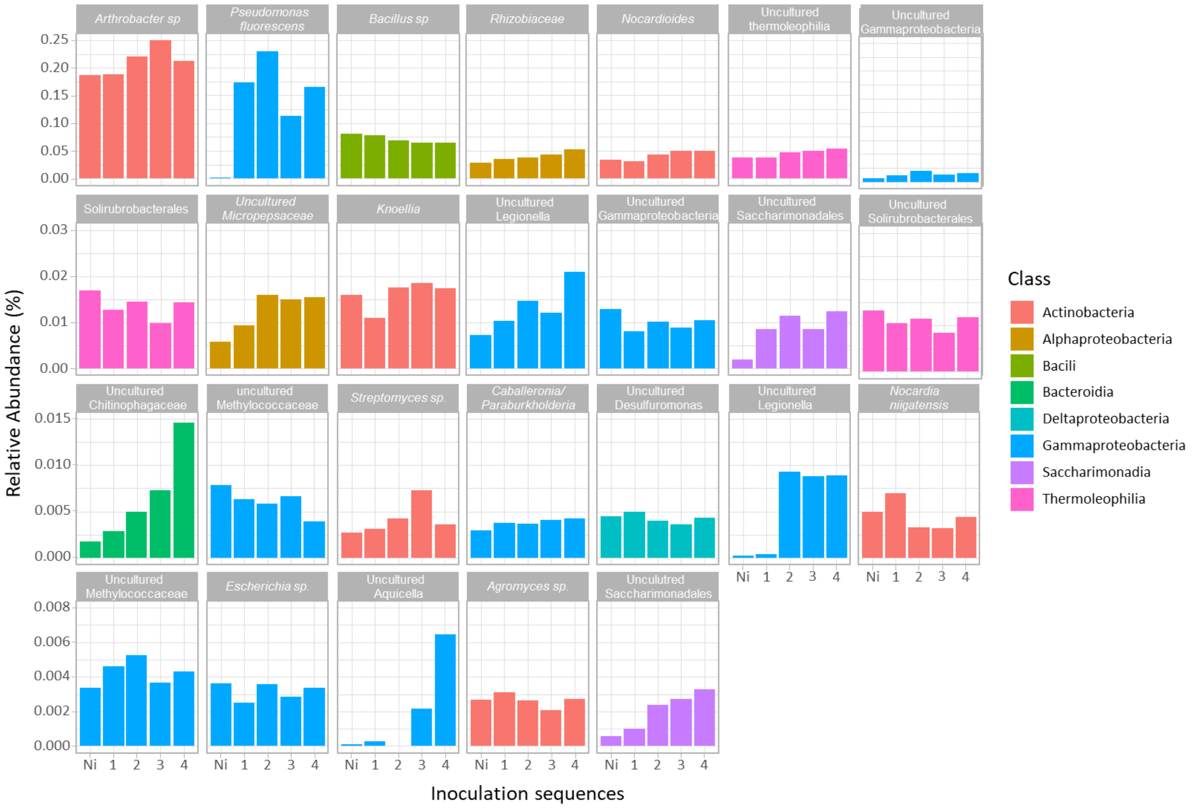

Supplementary 4: Abundance changes of OTUs. *Relative abundances of OTUs for which a significant difference is observed between the inoculation treatments and the NI control. Results are shown at week 10 for high density inoculation treatment (108 cfu.g dry soil^{-1}). Grey banners show each OTU taxonomy which are ordered by abundance from top to bottom. Y scale is not fixed.*
